# Perceptual vision training in non-sport-specific context: effect on performance skills and cognition in young females

**DOI:** 10.1038/s41598-019-55252-1

**Published:** 2019-12-10

**Authors:** Damiano Formenti, Marco Duca, Athos Trecroci, Leslie Ansaldi, Luca Bonfanti, Giampietro Alberti, Pierpaolo Iodice

**Affiliations:** 1 Department of Biomedical Sciences for Health, Università degli Studi di Milano, via Kramer 4/A, 20129 Milano, Italy; 2 Institute of Cognitive Sciences and Technologies, National Research Council, Via S. Martino della Battaglia 44, 00185 Rome, Italy; 3 Centre d’Etude des Transformations des Activités Physiques et Sportives (CETAPS) EA 3832, University of Rouen Normandy, Mont-Saint-Aignan, France

**Keywords:** Saccades, Decision, Attention, Perception

## Abstract

Although an increasing interest in vision training for sport performance, whether it may have a transfer to sport-specific skills and whether such transfer could be mediated by cognition remain open issues. To enlighten this point, we tested the effect of 6-weeks sport vision training programmes (requiring generic or volleyball-specific motor actions) in non-sport-specific context compared to a third group performing traditional volleyball training in sport-specific context. Fifty-one female volleyball players were randomly assigned to one of three groups. Before and after training period subjects were tested on accuracy of volleyball-specific skills and cognitive performance (clinical reaction time, executive control, perceptual speed). Accuracy of volleyball-specific skills improved after traditional volleyball training with respect to the vision training groups. Conversely, vision training groups improved cognitive performance (clinical reaction time, executive control and perceptual speed), as compared to traditional volleyball training group. Our results have shown that vision training in non-sport-specific context (both generic or with specific motor actions) improved cognitive performance, but seems to be less effective for improving sport-specific skills. These evidences suggest that environment in which exercises were performed plays a key role to improve perception and action in sport-specific skills, supporting the ecological approach to sport learning.

## Introduction

Statements as “eyes lead the body” or “do not take your eyes off the ball” are common expressions that highlight the role played by vision skills in sport performance. It is undoubted that vision plays a key role in the input of information used by perceptual-cognitive skills to perceive/represent the environment^1^. Imagine a match of team ball sport in which movements and actions must be continually adapted^2^. While the game develops in relatively unpredictable and changing contexts, players must not only maintain their focus on the ball but monitor the activities and positions of multiple players simultaneously (both opponents and teammates), planning and executing motor actions.

In this way, training of visual skills has aroused the interest of sport scientists and coaches. According to more influential sport learning theory, athletes need to develop not only physical and motor abilities but also visual and perceptual-cognitive skills^3,4^. The computational process and representational structures of environment, through a better visual information, improve the effectiveness of the chosen behaviour goal-directed^5^.

A recent study on the topic has shown that visual-motor abilities play a key role in performance and suggested that sensorimotor screenings may be a useful tool for player scouting^6^. Studies have also shown that elite athletes performed better in sport–specific cognitive tasks^7^, and this superiority has been supposed to have a transfer in general cognitive skills (non-sport–specific)^8^. Volleyball players would show highly flexible attention and superior executive control because volleyball training engages these abilities^9^. These abilities, commonly identified as “game intelligence” in sports, are denoted as executive functions (i.e., the cognitive processes that regulate thought and action)^10^.

Sport vision training, that uses stimuli in optometric tasks^11,12^, sport-specific videos or images^13^, or stroboscopic interruption of vision^14,15^, has been proposed under the idea that improving vision with oculomotor exercises, which might be associated with motor actions, would improve performance. For instance, Abernethy and Wood (2001) used generic stimuli (e.g., alphanumeric symbols, shapes, patterns and colours) presented to athletes in a form of painted charts or apparatuses^11^. Participants had to respond with simple ocular adjustments, which in some cases were combined with simple motor actions, such as pointing or touching targets^4^.

Although an increasing interest in vision training for sport performance, whether vision training would have a transfer to the field-sport setting remains unclear. Most of the intervention studies employed tasks based on optometric stimuli (e.g., hart charts and marsden ball) and on computer programs (e.g., D2 Dynavision and Eyeport) requiring simple ocular adjustments, and generic movements of hands as responses^11,16–19^. Schwab *et al*. (2012) reported that a group of hockey players who participated in a 6-weeks sport vision training programme improved on the same visual tasks used in the training, while no improvements were found on a transfer task. Furthermore, Abernethy and Wood (2001) demonstrated that generalized vision training improved some measures of visual skills, but such improvements were similar also for the control and placebo groups and were not reflected in field-based tennis transfer tests^11^.

The lack of evidences for supporting sport vision training efficacy to improve performance has been proposed to be related to methodological approaches resulting in a lack of ecological validity of the training stimuli^20^. As such, generic and automated nature of the motor actions required as response might have limited the potential effects on the sport performance. This lack of transfer seems to support the idea, proposed in ecological framework by Gibson, that perception and action have a direct circular relationship mediated by the information within external environment (i.e. affordances), rather than by internal representations^21^. It is therefore not possible to have learning except in the specific environment in which perception and action take place^22^. According to the Gibson’s theory, perception and action are conjoint in that they serve in reciprocal ways a mutual aim (i.e., reaching a goal), by detecting information that dynamically constrains action, and by controlling action that dynamically constrains perception^23^.

On the other hand, a recent study by Clark *et al*. (2012) found significant increases in several performance statistics (e.g., batting and hitting) in a season of a baseball team following the addition of non-sport–specific vision training with respect to the previous season^17^. Another study demonstrated that a non-sport–specific vision training period was able to induce significant improvement in a tennis field-based test^16^. These findings seem in contrast to the ecological dynamic framework, rather supporting the good-based models according to which perception (decision) and action should be considered separately. While such findings suggest a possible positive effect of generic vision training on the sport context, whether such training may have a direct transfer to sport-specific skills and whether such transfer could be mediated by cognition remain an open issue.

Vision training with eye exercises has been shown to positively affect cognitive functions^24,25^, which refer to subset of goal-directed, self-regulatory operations involved in the processes underling perception and action. These studies suggested the idea that vision training involves the ability to inhibit attention to task irrelevant or distracting stimuli. The ability to sustain attention involved a network of brain regions (prefrontal, temporal and parietal cortex) that exhibit high sensitivity to training stimuli^26^.

The positive effect of physical exercise on attentional skills and cognition is unanimously accepted in the literature in both adults and children^27^. Recently, studies have suggested that the effect of physical exercise on cognitive functions seems to be related to the exercise characteristics, specifically to the environmental context (dynamic and changing context, versus static and predictable context) in which exercises are performed^28^.

In the light of these considerations, we decided to study the effects on cognition of analytical exercises in a non-sport performance context with respect to exercises in a team-sport performance context, such as volleyball, which requires the perception and treatment of significant amount of information from external environment.

To study whether visual training would have an effect on sport-specific skills, we designed an experiment in which participants were assigned to three different 6-weeks training programmes. Two training programmes were characterized by analytic vision exercises combined with tasks requiring generic or sport-specific motor actions (both in non-sport performance context). The third group performed a traditional volleyball training programme in sport-specific environment and context (without analytic vision exercises). If perception of environment is based on visual space representations regarded as input analysing and computing, vision training could lead to an improvement in performance (transfer to sport-specific skills). Conversely, in accordance with an ecological view, the lack of transfer after vision training (with both generic or sport-specific tasks) together with improvements after traditional volleyball training should suggest that the expertise in sport could be more context-sensitive and consider jointly body and cognition.

A secondary outcome of the present study was to investigate whether different contexts in which physical exercises are performed, in relation to degrees of information-complexity and information-processing speed, would affect cognitive performance.

## Results

### Volleyball-specific skills

**Figure 1:**
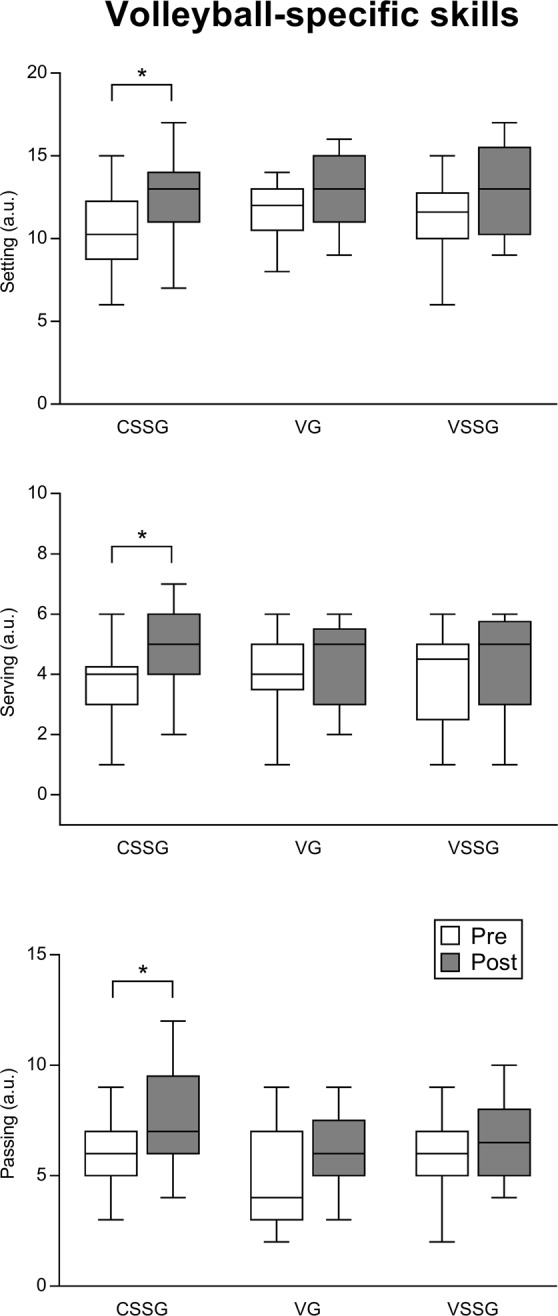
Effect of training intervention programmes on volleyball-specific skills (setting, serving, passing). Boxplot shows median and interquartile range, whiskers indicate the range. *p < 0.05. **p < 0.01. CSSG: context sport-specific group. VG: vision training group. VSSG: vision training sport-specific group. a.u.: arbitrary units.

The effect of training intervention programmes (context sport-specific group, CSSG; vision training group, VG; vision training sport-specific group, VSSG) on setting, serving and passing accuracy is shown in Fig. 1, together with pairwise comparisons. For setting accuracy, no significant interaction (time x group) was revealed (*F*_2,44_ = 0.21, *p* = 0.8), the main effect of group was not significant (*F*_2,44_ = 0.72, *p* = 0.48), whereas a significant main effect of time was found (*F*_1,44_ = 11.22, *p* = 0.0017). Specifically, LSD post hoc analysis showed that CSSG improved in setting accuracy from pre to post (*p* = 0.023, ES = 0.89, large), whereas VSSG (ES = 0.59, medium) and VG (ES = 0.77, medium) did not. For serving accuracy, no significant interaction (time x group) was revealed (*F*_2,44_ = 0.45, *p* = 0.63), the main effect of group was not significant (*F*_2,44_ = 0.06, *p* = 0.93), whereas a significant main effect of time was found (*F*_1,44_ = 8.94, *p* = 0.004). Specifically, CSSG improved in serving accuracy from pre to post (*p* = 0.02, ES = 0.63, medium), whereas VSSG (ES = 0.29, small) and VG (ES = 0.32, small) did not. For passing accuracy, no significant interaction (time x group) was revealed (*F*_2,44_ = 0.25, *p* = 0.77), the main effect of group was not significant (*F*_2,44_ = 2.97, *p* = 0.07), whereas a significant main effect of time was found (*F*_1,44_ = 8.51, *p* = 0.005). Specifically, LSD post hoc analysis showed that CSSG improved in passing accuracy from pre to post (*p* = 0.003, ES = 0.96, large), whereas VSSG (ES = 0.41, small) and VG (ES = 0.48, small) did not.

### Cognitive performance

**Figure 2:**
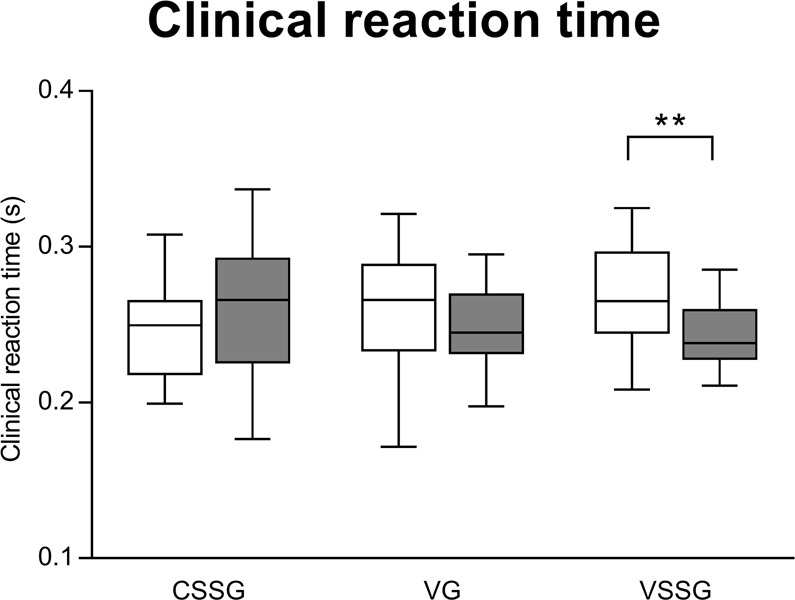
Effect of training intervention programmes on clinical reaction time. Boxplot shows median and interquartile range, whiskers indicate the range. **p < 0.01 CSSG: context sport-specific group. VG: vision training group. VSSG: vision training sport-specific group.

**Figure 3:**
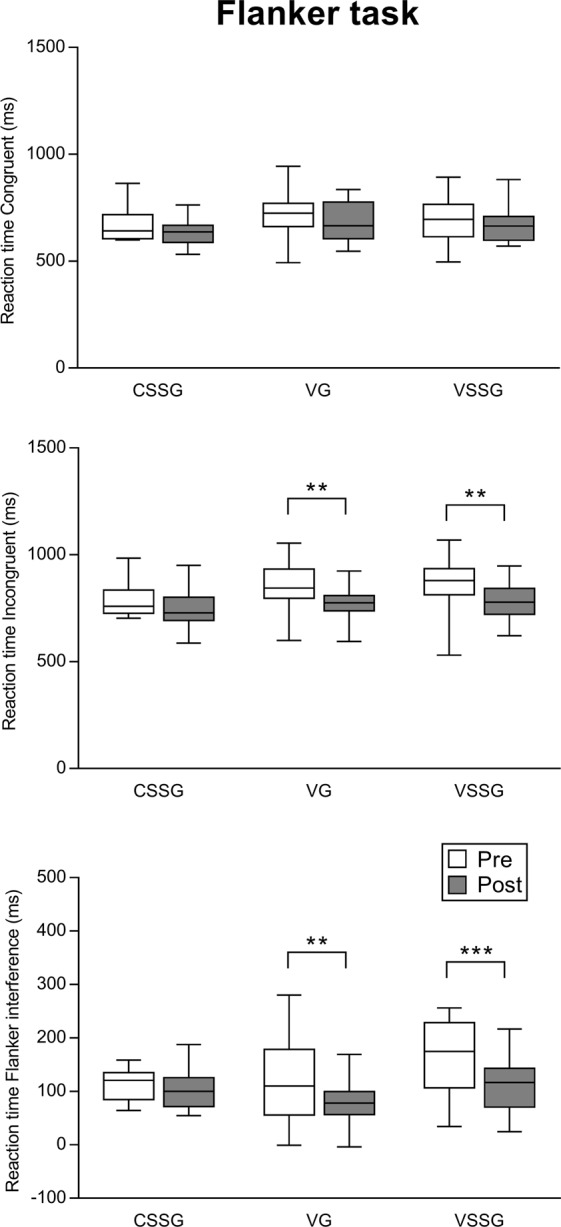
Effect of training intervention programmes on reaction time Congruent, reaction time Incongruent, and reaction time Flanker interference of the Flanker task. Boxplot shows median and interquartile range, whiskers indicate the range. **p < 0.01. ***p < 0.001. CSSG: context sport-specific group. VG: vision training group. VSSG: vision training sport-specific group.

**Figure 4:**
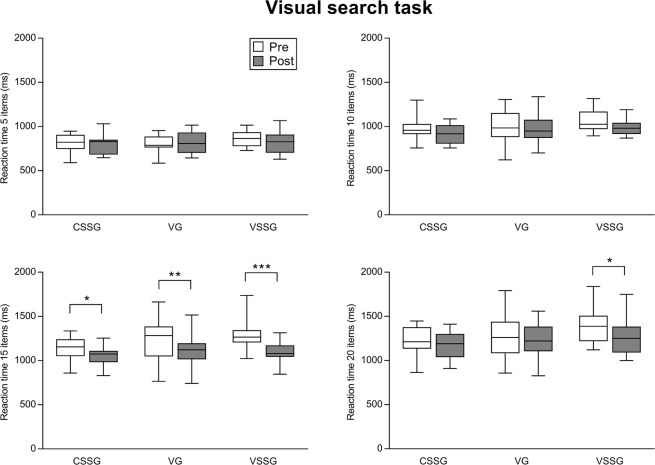
Effect of training intervention programmes on reaction time 5 items, reaction time 10 items, reaction time 15 items, reaction time 20 items of the Visual search task. Boxplot shows median and interquartile range, whiskers indicate the range. *p < 0.05. **p < 0.01. ***p < 0.001. CSSG: context sport-specific group. VG: vision training group. VSSG: vision training sport-specific group.

Overall, VSSG and VG improved their performance in clinical reaction time, Flanker task and Visual search task, while CSSG demonstrated less change (Figs. 2–4). The effect of training intervention programmes (CSSG, VG, VSSG) on clinical reaction time is shown in Fig. 2, together with pairwise comparisons provided by LSD post hoc analysis. A significant interaction (time × group) was revealed (*F*_2,44_ = 4.36, *p* = 0.018), implying that the training intervention programmes influences differently the clinical reaction time performance. Specifically, LSD post hoc analysis showed an improvement in reaction time performance for VSSG (*p* = 0.009, ES = 0.77, medium), but not for VG (ES = 0.29, small) and CSSG (ES = 0.48, small).

Figure 3 illustrates the effect of training intervention programmes on the variables of the Flanker task with LSD pairwise comparisons. For reaction time Congruent, no significant interaction (time x group) was found (*F*_2,44_ = 0.08, *p* = 0.42), the main effect of group was not significant (*F*_2,44_ = 1.58, *p* = 0.21), whereas there was a significant main effect of time (*F*_1,44_ = 5.69, *p* = 0.021), showing that overall VSSG (ES = 0.27, small), VG (ES = 0.40, small) and CSSG (ES = 0.39, small) increased their performance in reaction time Congruent from pre to post.

Similarly, for reaction time Incongruent, no significant interaction was revealed (*F*_2,44_ = 0.86, *p* = 0.42), the main effect of group was not significant (*F*_2,44_ = 1.6, *p* = 0.21), whereas there was a significant main effect of time (*F*_1,44_ = 23.58, *p* < 0.0001). Specifically, LSD post hoc analyses revealed that VSSG and VG significantly improved in reaction time Incongruent from pre to post (*p* = 0.001, ES = 0.61, medium, and *p* = 0.001, ES = 0.72, medium, respectively), whereas CSSG (ES = 0.48, small) did not.

For Flanker interference, no significant interaction (time x group) was found (*F*_2,44_ = 2.49, *p* = 0.078), the main effect of group was not significant (*F*_2,44_ = 2.36, *p* = 0.10), whereas there was a significant main effect of time (*F*_1,44_ = 18.95, *p* < 0.0001). Specifically, LSD post hoc analyses showed that VSSG and VG significantly improved in Flanker interference from pre to post (*p* < 0.001, ES = 0.74, medium, and *p* = 0.003, ES = 0.54, medium, respectively), whereas CSSG (ES = 0.26, small) did not.

Figure 4 shows the effect of training intervention programmes on the variables of the Visual search task with LSD pairwise comparisons. For reaction time 5 items, no significant interaction (time × group) was revealed (*F*_2,44_ = 0.93, *p* = 0.401), and the main effect of group (*F*_2,44_ = 0.93, *p* = 0.39) and time (*F*_1,44_ = 1.05, *p* = 0.31) were not significant as well.

For reaction time 10 items, no significant interaction (time × group) was found (*F*_2,44_ = 0.21, *p* = 0.805), the main effect of group was not significant (*F*_2,44_ = 1.36, *p* = 0.26), whereas a significant main effect of time was found (*F*_1,44_ = 7.83, *p* = 0.007), showing that overall VSSG (ES = 0.54, medium), VG (ES = 0.20, small) and CSSG (ES = 0.41, small) increased their performance in reaction time 10 items from pre to post.

Similarly, for reaction time 15 items, no significant interaction (time x group) was revealed (*F*_2,44_ = 1.75, *p* = 0.18), the main effect of group was not significant (*F*_2,44  _=2.06, *p* = 0.13), whereas a significant main effect of time was found (*F*_1,44_ = 37.93, *p* < 0.0001). Specifically, LSD post hoc analysis showed that VSSG, VG and CSSG improved their performance in reaction time 15 items from pre to post (*p* < 0.0001, ES = 1.16, large for VSSG, *p* = 0.003 ES = 0.49, small for VG, *p* = 0.017, ES = 0.72, medium for CSSG).

For reaction time 20 items, no significant interaction (time × group) was revealed (*F*_2,44_ = 0.65, *p* = 0.52), the main effect of group was not significant (*F*_2,44_ = 2.10, *p* = 0.13), whereas a significant main effect of time was found (*F*_1,44_ = 5.64, *p* = 0.021). Specifically, LSD post hoc analysis showed that VSSG improved in reaction time 20 items from pre to post (*p* = 0.026, ES = 0.65, medium), whereas VG (ES = 0.15, small) and CSSG (ES = 0.36, small) did not.

## Discussion

Many contemporary studies have assessed the effect of visual training programme on visual skills and sport performance, by varying experimental parameters such as stimuli or contexts^11,16–18^. These and other studies have shown that visual perception skills and cognitive performance are improved after a vision training programme^24^.

Despite this progress, relatively little is known on how generic visual perception skills affect sport performance. In particular, if an increase in perception skills independently from sport action environment could affect the sport-specific skills and sport-related cognitive abilities is unclear.

Two notable results were reported in this study. First, by manipulating different visual stimuli and context (generic vision stimuli with generic motor actions, generic vision stimuli with specific motor actions, and sport-specific vision stimuli in sport-specific context), we are able to improve performance skills only after training in sport-specific context. Conversely, visual training with both generic and sport-specific motor actions did not permit a transfer to performance skills. Second, both vision training programmes (with tasks requiring generic or sport-specific motor actions) improved cognitive performances (i.e. clinical reaction time, executive control and perceptual speed).

To investigate the role of vision training with respect to a traditional training on sport-specific skills, we tested fifty-one volleyball players on their abilities to perform sport-specific skills on accuracy of passing, setting, and serving^29^. The main effect of time revealed general improvements in the accuracy of all volleyball-specific skills. However, the post-hoc comparisons revealed that the traditional volleyball training group (CSSG) was the only significant (see Fig. 1 for more details). Altogether, these findings provided evidence that a traditional volleyball training seems to be superior than vision training for improving sport-specific skills. These evidences seem to suggest that environment in which exercises were performed plays a key role to improve perception and action in performance skills^22^.

Our results support the idea proposed by the ecological dynamic theory that skill acquisition is performer-environment related^30^. Information sources from a particular performance context specify the actions that performers need to make, by *affording* opportunities to act^31^. The ability of athletes to identify the specific variables and to actualise the affordances for action supports successful performance in specific contexts. This process is based on the creation and subsequent refinement of information-movement coupling to achieve adaptive behaviour. The process of attending to a more functional perception of variables, or combination of variables, occurs by training sport-specific skills in a congruent context that permits to improve attention or perceptual attunement^21^.

The current study applied both generic and sport-specific vision training methods to explore how performing a sport action during vision exercises would affect the transfer of vision skills on sport performance. Our data indicate that performing a sport action, in a non-sport-specific context, seems not to induce an augmentation in learning transfer effect on sport performance^32^. These results point to the conclusion that organism-environment coupling is a fundamental element of sport-skills learning. During motor learning, the intentions of an individual needs to correspond to a task goal, and each learner is challenged to coordinate the abundant perceptual and motor system degrees of freedom in achieving a specific task goal. In the learning process, calibration and recalibration are necessary to maintain functionality of perception^33^.

It is worth noticing that the testing battery used to test volleyball-specific skills^29^, albeit in a sport-specific context, includes the assessment of volleyball-specific skills in an isolated domain. Since individuals’ ability to execute skills successfully after making a decision in a real-field setting (game-specific performance) is of great importance^13^, future studies should also use real-time monitoring approaches (i.e., match analysis, scouting) to assess perceptual-cognitive skills such as decision making and anticipation. This would also allow to use a real competition setting to test the presence of an effective transfer of sport vision training on game-specific performance.

Concerning cognitive variables, our results seem to support the idea that eye exercises are able to improve cognitive performance^24,25^. In particular, these enhancements may be related to the fact that vision training involves the ability to inhibit attention to distracting stimuli, by activating the brain regions responsible of cognitive functions.

Improvements in computer-based tasks performances (i.e., Flanker and Visual search tasks) were found for VSSG and VG. In the Flanker task, no significant improvement was found in reaction time Congruent for neither group. On the contrary, although a lack of time x group interaction, the main effect of time was significant: VG and VSSG, but not CSSG, improved their pre-post performance by the post-hoc analysis in reaction time Incongruent and reaction time Flanker interference (Fig. 3). Incongruent condition requires great attentional control to filter potentially misleading distractors (i.e., flankers) reflecting incorrect behavioural responses^34^. Our results support the findings of a previous study showing that physical activity appears related to performance requiring high level of attentional control (i.e., incongruent conditions that contain distracting information)^35^.

The positive changes in executive control of sport vision training groups (VSSG and VG) were accompanied by an improvement in perceptual speed, assessed by the Visual search task (Fig. 4). The significant main effects of time found for reaction time 10 items, 15 items and 20 items suggest that there was an overall improvement in perceptual speed performance, regardless of the type of training. However, the post-hoc analysis revealed that the three groups improved only in reaction time 15 items, whereas VSSG improved also in reaction time 20 items. This confirms the notion that tasks requiring greater amounts of interference control (Incongruent condition of the Flanker task, and 15 and 10 items of the Visual search task) seem to be superior for highlighting possible improvements related to physical exercise^35^.

It should be acknowledged that, although the significant main effect of time for both the Flanker task and the Visual search task, no significances were found in the post-hoc comparisons for CSSG. The lack of significant improvement in the cognitive performance of the CSSG appears to reject a theoretical framework according to which activities in a dynamic and changing context might have superior benefit for cognition than activities in static and predictable context^28^. This points to the conclusion that oculomotor exercises involving brain regions responsible of cognitive functions, albeit in a non-sport–specific context, seem to play a major role for enhancing cognitive performance. There is evidence that the frontal eye fields, an oculomotor region of the premotor cortex, is of primary importance during voluntary eye movements^36^. Accordingly, studies proposed an underlying neural mechanism that facilitates the improvement of visual attention following vision training with eye exercises. Common functional regions of the cortex were activated during tasks of shifting visual attention and eye movements, which include the superior temporal, intraparietal, and portions of the precentral sulcus, and medial frontal gyrus^37^. The synchronous activation of these brain regions during vision training has been suggested to be sufficient for inducing short-term plasticity^24^.

The notion of the link between vision training and cognition was also supported by a recent longitudinal study showing that 12 sessions of vision training enhanced cognitive performance on attention and memory tasks^24^. These enhancements were supposed to be mediated by short-term changes in neural activity and/or short-term plasticity of frontal and parietal regions, which are responsible for visual attention, preparatory motor signalling of the visuomotor system, and working memory^36–41^.

Similar to computer-based tasks, we found that VSSG enhanced performance in the clinical reaction time. Although the pre-post comparison of VG was not significant, overall performing vision training seems to induce improvements in clinical reaction time. This is supported by the significant time x group interaction, revealing that VSSG tended to enhance clinical reaction time with respect to VG and CSSG (Fig. 2). An explanation to the improvement of VSSG in the clinical reaction time can be related to the feature of vision training of combining vision stimuli with motor actions to be performed as rapidly as possible. The clinical reaction time test (i.e., a clinical measure of simple reaction time) is a task requiring the subject to focus on the weighted disk, catching the apparatus as rapidly as possible once released by the examiner^42^. Generalizing, subjects are required to rapidly identify and recognize the fall of the apparatus, and to decide on catching action (e.g., if, when and how to act). It is possible that sport vision training improved clinical reaction time by acting on the role of visual attention in the perception-action cycle and eye-hand coordination^43^.

The current study presents limitations that should be acknowledged. First, as executive functions develop from early childhood through adolescence into adulthood^44^, we cannot fully exclude the possibility that the pre-post improvements of the three groups regardless of the type of training (main effect of time), might be related - at least partially - to the natural development of executive functions. Finally, we put in evidence that our findings cannot be surely extended also to male subjects. Cognitive abilities are influenced by many variables, such as age^45^ and sex^46^. Thus, further studies will be addressed to compare the effect of vision training on cognition and skill performance in male and female subjects, as well as its response in relation to different ages. Moreover, further researches should investigate whether the improvements related to vision training could be also maintained after a retention period, possibly for long-term effects.

In conclusion, our experiment demonstrated that vision training (in non-sport-specific context), despite the improvements in cognitive tasks, seems to be less effective than traditional volleyball training for improving sport-specific skills. Analytics practices drills for acquiring general vision skills in time reaction or visual research task are reductionist and involve vision tasks repetitions in which the frequency or volume of practice is emphasised.

In other words, our results showed that the continuous performer-environment interactions emphasise adaptive movement variability in performing sport-specific skills. In team sports, inter-personal coordination is grounded on the players’ ability to identify and calibrate specific information for the action capabilities of teammates and opponents. Designing practice tasks that simulate performance environments is an important challenge for sport scientists and practitioners. Moreover, investigating whether conditioned or small-sided games might be more functional in adaptive learning process that characterises sport rest a fundamental issue in sport research.

## Methods

### Participants

Fifty-one female young volleyball players were recruited for the study. They were randomly divided into three groups for assigning participants to vision training group (VG), vision training sport-specific group (VSSG), and context sport-specific group (CSSG). Seventeen players were allocated in the VG (age: 10.95 ± 0.71 years; body mass index: 17.83 ± 1.54 kg m^−2^; maturity offset: −2.6 ± 0.64). Seventeen players were allocated in the VSSG (age: 11.13 ± 0.77 years; body mass index: 17.89 ± 2.65 kg m^−2^; maturity offset: −2.71 ± 0.65). Seventeen players were allocated in the CSSG (age: 11.2 ± 0.73 years; body mass index: 18.89 ± 2.19 kg m^−2^; maturity offset: −2.6 ± 0.6). Unfortunately, one participant of the VSSG and three participants of the CSSG dropped out prior to conclude the study for external reasons. No significant differences were found between groups for age, body mass index and maturity offset. All volleyball players had training history of at least 4 years, which included mini volleyball game models within the first 2 years. Mini volleyball game is a game played by 4 people that masters basic techniques of volleyball. Then, more advanced forms of game and volleyball-related skills have been introduced within the following 2 years together to the first form of competition games. All players and their parents were informed about the purpose and experimental protocol of the study. Parents or legal guardians provided the written informed consent before the investigation. In accordance with the Declaration of Helsinki, the study was approved by the Ethics Committee of the University of Milano.

## Experimental design

**Figure 5:**
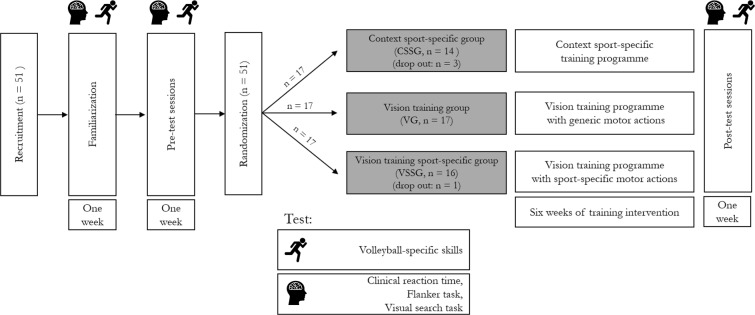
Overview of the experimental protocol.

Two testing sessions were scheduled within one week before the start of the training period (pre) and after six weeks of training (post). The first testing session aimed to collect anthropometric variables and to assess cognitive performance with reaction time and two cognitive tests reflecting perceptual speed and executive control. The second testing session aimed to assess volleyball-specific skills. After the six weeks of training, participants completed post-test sessions that were identical to the pre-test sessions. Thus, the whole study lasted 8 weeks for each participant. The subjects were instructed to refrain from strenuous physical activity in the two days before the testing sessions. Participants underwent two preliminary sessions before the pre-test sessions to familiarize with the testing procedure. A schematic representation of the experimental design is shown in Fig. 5.

### Cognitive performance tests

#### Reaction time

Reaction time was assessed using the clinical reaction time test^42^. During clinical reaction time testing procedure, participants sat with their dominant forearm on a table, with their open hand at the edge of the table. The examiner suspended the clinical reaction time apparatus vertically in a way that the weighted disk was aligned with the top of the participant’s open hand. The examiner released the apparatus at predetermined random time intervals (from 4 to 15 seconds) and the participant had to catch it as quickly as possible. The participant was requested to maintain his gaze on the weighted disc. Gazing at examiner’s hand was not allowed. The distance - in centimeters - from the top of the disk to the most superior part of the participant’s hand was recorded. This distance was converted to clinical reaction time, in milliseconds, using the equation of time needed for an object to fall a given distance. Each participant performed eight trials, and the mean value among these eight trials was considered for subsequent analysis.

#### Executive control

Executive control was measured using a modified arrow version of the Flanker task^47^. Participants had to respond to the direction of a left or right pointing target arrow while having to ignore flanking arrows that pointed either in the same or the opposite direction. Two flanking arrows that had to be ignored were presented to the left and to the right of the target arrow. This modified Flanker task included two response conditions. The congruent condition consisted of a trial in which both the target arrow and the flanking arrows pointed in the same direction (left: <<<<< or right: >>>>>). The incongruent condition consisted of a flanking arrow that pointed in the opposite direction of the target arrow (<<><< or >><>>). Participants were requested to press the button A of the keyboard when the target arrow pointed to the left, and the button L when the target arrow pointed to the right. Participants underwent two blocks of 50 trials each. The 100 trials presented in this task were distributed equally among the two experimental conditions and were presented in a random order. Participants had 2 s to provide their response to the target arrow. Participants performed a practice block of 10 trials before the two 50 trials blocks to ensure they understood the directions of the task. Only correct responses were included in the outcome variables. Three variables were extracted and considered for analysis: mean reaction time in congruent conditions (reaction time Congruent), mean reaction time in incongruent conditions (reaction time Incongruent), and Flanker interference. Flanker interference was calculated by subtracting reaction time of congruent trials from reaction time of incongruent trials. Flanker task was programmed using the experimental software Psytoolkit^48^.

#### Perceptual speed

Perceptual speed was assessed by Visual search task^49^. The target stimulus was an orange letter T, and distractors stimuli were blue T and an upside-down orange T. Participants had to press the space button of the keyboard if the target stimulus was present among distractor stimuli and withhold a response if it was not present. The number of items for each trial among which target stimulus might be present was 5, 10, 15 and 20, which were randomized. Half the search trial had no target stimulus. There were 100 trials divided into two blocks of 50 trials each. Each trial started with a fixation point of 100 ms, followed by a delay of 400 ms, followed by the search display. Participants had to respond within maximally 4 s. As feedback, in case participants did not respond to the presence of a target stimulus, the target location would be highlighted for 2 s to identify the missed target. Similarly, in case participants responded in the absence of a target stimulus, they were informed about wrong response for 2 s. Participants performed a practice block of 10 trials before the two 50 trials blocks to ensure that they understood the directions of the task. Only correct responses were included in the outcome variables. Four variables were extracted and considered for analysis: mean reaction time in 5 items displays (reaction time 5 items), mean reaction time in 10 items displays (reaction time 10 items), mean reaction time in 15 items displays (reaction time 15 items) and mean reaction time in 20 items displays (reaction time 20 items). Visual search task was programmed using the experimental software Psytoolkit^48^.

### Volleyball-specific skills

Volleyball-specific skills were assessed in a sport-field setting. Participants were tested on accuracy of passing, setting, and serving skills in an indoor stadium using a field-based testing battery proposed by Gabbet and Georgieff^29^. Participants were familiarized with the testing procedure before the beginning of the study. Two digital cameras (FDR1000V, Sony, Tokyo, Japan), positioned approximately 5 m from the player, were used to film each skill. In the case of setting, players were filmed from the side and behind of the player, while serving and passing skills were filmed from the side and front of the player. The participants’ accuracy was based on their ability to hit specific targets. Participants performed six trials of each skill. For each skill, the overall score used as dependent variable was the sum of points of the six trials.

As this testing battery was developed for junior players (15.5 ± 1.0 years), for setting skill we decided to reduce the distance between the target (positioned next to the net) and the setting player from 5.5 m – as proposed by Gabbett and Georgieff (2006) - to 4 m. This was a necessary modification because 5.5 m was a too large distance for our participants (11.09 ± 0.73 years) to perform a good setting reaching the target. A coach, positioned approximately 5 m from the setting player, threw an overhead pass to the setting player. Players had to set the ball to the circular target (80 cm in diameter). Players who successfully set the ball through the target were awarded 3 points. Balls that hit the outside edge of the target were awarded 2 points. Players who set the ball outside of the circular target, but within 2.3 m from the net (and therefore 1.5 m from the target) were awarded 1 point. Balls that did not reach the target areas were awarded 0 points.

Serving skill was determined as the ability of players to serve in the entire court (9 m width) from a service position, with no specific service targets (1 point for a ball within the court, 0 point for a ball out of the court).

Passing skill was evaluated by determining players’ ability to return a pass to a target (1.6 m long and 2.3 m wide) positioned at the net, 2 m from the right-hand sideline. This target was chosen because was the approximate position of the setter during a match. A coach, positioned in the service position, approximately 1 m above the ground and 10 m from the receiving player, threw an overhead pass to the receiving player. Players were required to pass the ball to another player standing with arms extended above their head (setter), in the target area. Players who successfully passed the ball to the player in the target area were awarded 2 points. A second target area was created for passes that did not reach the main target area (extended from the right-hand sideline, 3 m long and 4.1 m wide), but would be likely to reach another player in a match situation. A pass reaching the second target area was awarded 1 point, whereas a pass that did not reach either of the target areas was awarded 0 points. For a schematic representation of the testing battery please refer to a previous study^50^.

### Training interventions

Each training session lasted 90 min. It was composed by four parts: warm-up (10 min), training intervention (30 min), physical exercises (20–30 min), cool-down phase (10 min). Physical exercises phase was based on training physical components of volleyball, such as agility and jumping ability. The cool-down phase mainly included stretching and mobility exercises.

**Figure 6:**
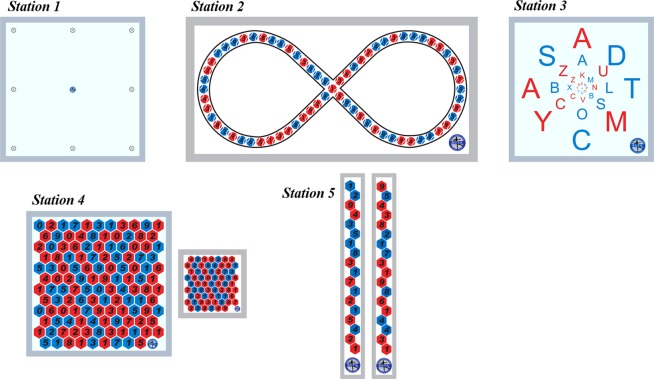
Representation of boards (S.V.T.A. method©, Carmagnola, Italy) utilized for each station within a vision training session for VG (vision training group) and VSSG (vision training sport-specific group).

A sport science graduate and volleyball coach constantly supervised the entire execution of all training sessions. The training programme of VG and VSSG was designed with the following equipment: S.V.T.A. training kit premium (S.V.T.A. method^©^, Carmagnola, Italy). These training aimed to combine visual skills as peripheral vision, saccadic eye movements, accommodation and vergence skills, with motor actions to be performed as rapidly as possible. Each training session was structured as circuit training so that each participant of each group exercised all the five different stations for 6 min. Each station was characterized by different boards (Fig. 6), fixed on a wall at 1.5 meter from ground level, with colours, letters and numbers as targets. VG and VSSG programmes were structured in three levels of increased difficulty: level 1 from sessions 1 to 4, level 2 from sessions 5 to 8, and level 3 from sessions 9 to 12.

#### CSSG training programme

The CSSG performed a traditional volleyball training based on simple repetitive exercises of technique of volleyball skills in a sport-specific environment and context (without analytic vision exercises). Training intervention was based on a combination of technical skills and instructional coaching, matched with skill-based games to facilitate learning. Specifically, it was based on small sided games (e.g., 3 vs. 3 or 5 vs. 5) and repetitive exercises reflecting specific game situations with both teammates and opponents.

#### VG training programme

VG performed the following analytic vision training exercises combined with generic motor actions in a non-sport performance context:

Station 1: A square board (50 cm) with nine points at equal distance (15 cm each) was fixed on a wall. Participants were requested to move their gaze on external points in clockwise and anticlockwise direction, maintaining their focus on the central point. Level 1: 1 m from the wall, bipodalic standing. Level 2: 0.75 m from the wall, monopodalic standing. Level 3: 0.5 m from the wall, monopodalic standing on skimmy cushion.

Station 2: A rectangular board (25 high and 50 wide cm) with an infinity picture was fixed on a wall. The infinity profile was composed by a sequence of randomized red and blue points. Participants were requested to move their gaze from point to point following the infinity profile (one rounds for direction) pronouncing the colour of the point identified. Level 1: 1 m from the wall. Two additional boards were positioned on the floor on the right and left side of the participant, 50 cm from the standing position, one red and one blue, respectively. Participants were requested to touch with foot the board of the corresponding colour. Level 2: 0.75 m from the wall. As level 1, but running toward the corresponding colour, positioned 2 m apart from the starting position, and coming back. Level 3: 0.5 m from the wall. The same as level 2, but the starting position was a monopodalic standing on skimmy cushion.

Station 3: A square board (50 cm) with four concentric circles composed by a sequence of randomized red and blue letters was fixed on a wall. Participants were requested to move their gaze from letter to letter, pronouncing the letter identified while maintaining their focus on the central point (small black point in the centre of the board). After the first round performed on the external circle in both clockwise and anticlockwise direction, the following round was performed on the inner circle, and so on. Level 1: 1 m from the wall. Level 2: 0.75 m from the wall. In bipodalic standing on skimmy cushions. Level 3: 0.5 m from the wall. The same as level 2, but in monopodalic standing on skimmy cushion.

Station 4: A square board (50 cm) depicting a honeycomb structure, composed by 83 cells, was fixed on the wall. Cells were red or blue, each containing a random numerical digit from 0 to 9. Participants held in hands a square board (12 cm) with a honeycomb structure depicted on, composed by 67 black coloured numerical digits inside grey cells (from 0 to 9, randomized). Participants were requested to read the numerical digit on the board in hands (beginning from the first row, from left to right) and localize the same numerical digit on the board on the wall (beginning from the first row, from left to right). Level 1: 1 m from the wall. Two boards were positioned on the floor on the right and left side of the participant, 2 m from the standing position, one red and one blue, respectively. After identifying the colour of the numerical digit, participants were requested to run toward and touch with foot the board of the corresponding colour. Level 2: 1.5 m from the wall. Two additional boards were positioned immediately below the board on the wall, one red and one blue, interspersed by 50 cm. After identifying the colour of the numerical digit, participants had to touch with hand the board of the corresponding colour. Level 3: 2 m from the wall. Two cones were positioned on the floor on the right and left side of the participant, 2 m from the standing position on each side, one red and one blue, respectively. After identifying the colour of the numerical digit, participants had to run around the cone of the corresponding colour.

Station 5: Two boards (50 cm high and 6 cm wide) with black coloured numerical digits were fixed on the wall vertically, 1 m apart. Red and blue cells contained a numerical digit from 0 to 9. Participants were requested to read digits shifting gaze from one board to the other. First beginning from up-left, then from up-right, and finally from down-left and down-right. Level 1: 2 m from the wall. While shifting gaze from one board to the other, if the participants read an even number, they had to lunge forwards, otherwise if the number were odd, they had to lunge backwards. Level 2: 2 m from the wall. Four additional boards were positioned on the floor immediately ahead from the standing position, each one with a numerical digit from 1 to 4. Participants were requested to read the numerical digit on the wall board and touch with the foot the board on the floor of the corresponding numerical digit. Level 3: 2 m from the wall. The same as level 2 but jumping with both lower limbs on the corresponding numerical digit instead of touching with foot.

#### VSSG training programme

VSSG performed the same exercises as VG, with one main difference: motor actions were not generic but volleyball-specific, also with the use of the volleyball.

Station 1: The same as VT programme, but with the addition of setting a volleyball. Level 1: 1 m from the board. While performing the vision exercise, participants had to set above their head with the volleyball. Level 2: 0.75 m from the board, monopodalic standing. While performing the vision exercise, participants had to set above their head with the volleyball. Level 3: 0.5 m from the board, monopodalic standing on skimmy cushion. While performing the vision exercise, participants had to set above their head with the volleyball.

Station 2: The same as VT programme, but with volleyball specific movements. Level 1: 1 m from the wall. Two additional boards were positioned on the floor on the right and left side of the participant, 50 cm from the standing position, one red and one blue, respectively. Participants were requested to lunge laterally toward the board of the corresponding colour. Level 2: 0.75 m from the wall. As level 1, but shuffling toward the corresponding colour, positioned 2 m apart from the starting position, and coming back. Level 3: 0.5 m from the wall. Similar to level 2, but the starting position was a monopodalic standing on skimmy cushion.

Station 3: The same as VT programme, but with the use of the volleyball. Level 1: 1 m from the wall. While performing the vision exercise, participants were requested to juggle the volleyball. Level 2: 0.75 m from the wall. In bipodalic standing on skimmy cushions, while performing the vision exercise, participants were requested to juggle the volleyball. Level 3: 0.5 m from the wall. The same as level 2, but in monopodalic standing on skimmy cushion.

Station 4: Similar to VT programme, participants were requested to read the numerical digit on the board in hands (beginning from the first row, from right to left) and localize the same numerical digit on the board on the wall (beginning from the first row, from right to left). Level 1: 1 m from the wall. Two cones were positioned on the floor on the right and left side of the participant, 2 m from the standing position, one red and one blue, respectively. After identifying the colour of the numerical digit, participants had to lunge laterally towards the cone of the corresponding colour. Level 2: 1.5 m from the wall. Two additional boards were positioned immediately below the board on the wall, one red and one blue, interspersed by 50 cm. After identifying the colour of the numerical digit, participants had to pass (preceded by a preparatory setting) toward the board of the corresponding colour. Level 3: 2 m from the wall. Two boards were positioned on the floor on the right and left side of the participant, 50 cm from the standing position on each side, one red and one blue, respectively. After identifying the colour of the numerical digit, participants had to do a rolling dive toward the board of the corresponding colour.

Station 5: Similar to VT programme but with the addition of volleyball. Level 1: 2 m from the wall. While shifting gaze from one board to the other, if the participants read an even number, they had to complete a set against the wall, otherwise if the number were odd, they had to execute a pass. Level 2: 2 m from the wall. While shifting gaze from one board to the other, participants were requested to do a volleyball specific action if the last read digit were 1, 2, 3, 4. 1 = setting against the wall; 2 = passing against the wall; 3 = serving underhand; 4 = spiking. Level 3: 2 m from the wall. The same as level 2, but participants were requested to direct the volleyball in each fundamental skill toward a corresponding coloured board positioned on the wall.

### Statistical analysis

Data are expressed as mean ± SD. The normality of the distribution of the data was checked using the D’Agostino Pearson test and visual inspection. No significant difference between groups – by one-way analysis of variance (ANOVA) - was found for each variable in pretraining test evaluation. A two-way (time and group) ANOVA with repeated measures on one factor (time) was used to investigate the effect of the training intervention on each variable. Least significant difference (LSD) post-hoc analyses were used to compare pairs of means. Effect sizes were calculated to assess the magnitude of the difference^51^. Cohen’s d values between 0.20 and 0.49 indicated *small* effect, values between 0.50 and 0.79 indicated a *medium* effect, and values of 0.80 and above indicated a *large* effect size. The level of significance was set at *p* ≤ 0.05. Statistical analysis was performed using SPSS 21.0 for windows (IBM SPSS Statistics, Inc., New York, NY, USA).

## Data Availability

The datasets generated during the present study are available from the corresponding author on reasonable request.
